# An unknown solvate of 1-(2,4-di­chloro­benz­yl)-4-[(4-methyl­phen­yl)sulfon­yl]piperazine

**DOI:** 10.1107/S160053681301012X

**Published:** 2013-04-24

**Authors:** S. Sreenivasa, K. E. ManojKumar, H. C. Anitha, P. A. Suchetan, B. S. Palakshamurthy, Yenagi Jayashree, J. Tonannavar

**Affiliations:** a Department of Studies and Research in Chemistry, Tumkur University, Tumkur, Karnataka 572 103, India; b Center for Advanced Materials and Department of Chemistry, Tumkur University Tumkur, Karnataka 572103, India; c Department of Studies and Research in Chemistry, U.C.S, Tumkur University, Tumkur, Karnataka 572 103, India; d Department of Studies and Research in Physics, U.C.S, Tumkur University, Tumkur, Karnataka 572 103, India; e Department of Physics, Karnatak University, Dharwad, Karnataka 580 003, India

## Abstract

In the title compound, C_18_H_20_Cl_2_N_2_O_2_S, the piperazine ring adopts a chair conformation. The dihedral angle between the sulfonyl-bound benzene ring and the best-fit plane through the six non-H atoms of the piperazine ring is 72.22 (12)°; those between the di­chloro­benzene ring and the sulfonyl and piperazine rings are 2.44 (13) and 74.16 (2)°, respectively. In the crystal, mol­ecules are connected through weak C—H⋯O inter­actions into a hexa­meric unit generating a *R*_6_^6^(60) motif in the *ab* plane. The mol­ecules are also connected into *C*(4) chains through weak C—H⋯N inter­actions. The solvent used to grow the crystal was a mixture of di­chloro­methane and methanol, but the resulting electron density was uninter­pretable. The solvent contribution to the scattering was removed with the SQUEEZE routine in *PLATON* [Spek (2009 ▶). *Acta Cryst*. D**65**, 148–155]. The formula mass and unit-cell characteristics do not take into account the disordered solvent.

## Related literature

For similar structures, see: Sreenivasa *et al.* (2013*a* ▶,*b* ▶).
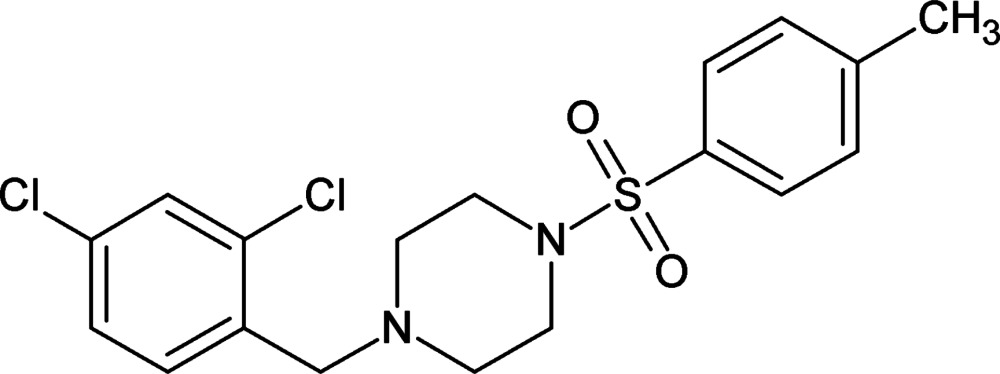


## Experimental

### 

#### Crystal data

C_18_H_20_Cl_2_N_2_O_2_S
*M* *_r_* = 399.32
Trigonal, 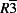

*a* = 28.2896 (5) Å
*c* = 13.3041 (3) Å
*V* = 9220.8 (3) Å^3^
*Z* = 18
Mo *K*α radiation
μ = 0.43 mm^−1^
*T* = 296 K
0.31 × 0.23 × 0.19 mm

#### Data collection

Bruker APEXII diffractometer
15194 measured reflections
3596 independent reflections
2637 reflections with *I* > 2σ(*I*)
*R*_int_ = 0.033
3608 standard reflections every 22 reflections intensity decay: 1.0%

#### Refinement

*R*[*F* ^2^ > 2σ(*F* ^2^)] = 0.038
*wR*(*F* ^2^) = 0.109
*S* = 0.95
3596 reflections
227 parameters
H-atom parameters constrained
Δρ_max_ = 0.20 e Å^−3^
Δρ_min_ = −0.25 e Å^−3^

### 

Data collection: *APEX2* (Bruker, 2009 ▶); cell refinement: *APEX2* and *SAINT-Plus* (Bruker, 2009 ▶); data reduction: *SAINT-Plus* and *XPREP* (Bruker, 2009 ▶); program(s) used to solve structure: *SHELXS97* (Sheldrick, 2008 ▶); program(s) used to refine structure: *SHELXL97* (Sheldrick, 2008 ▶); molecular graphics: *Mercury* (Macrae *et al.*, 2008 ▶); software used to prepare material for publication: *SHELXL97*.

## Supplementary Material

Crystal structure: contains datablock(s) I, global. DOI: 10.1107/S160053681301012X/tk5213sup1.cif

Structure factors: contains datablock(s) I. DOI: 10.1107/S160053681301012X/tk5213Isup2.hkl

Supplementary material file. DOI: 10.1107/S160053681301012X/tk5213Isup3.cml

Additional supplementary materials:  crystallographic information; 3D view; checkCIF report

## Figures and Tables

**Table 1:** Hydrogen-bond geometry (Å, °)

*D*—H⋯*A*	*D*—H	H⋯*A*	*D*⋯*A*	*D*—H⋯*A*
C18—H18⋯O2^i^	0.93	2.70	3.575 (3)	157
C17—H17⋯N2^ii^	0.93	2.70	3.485 (3)	143

Symmetry codes: (i) 
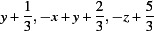
; (ii) 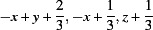.

**Fig. 1.**
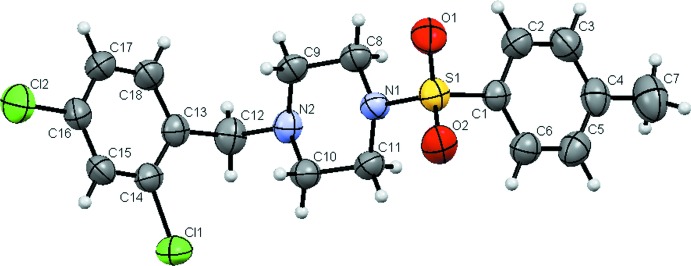
Molecular structure of the title compound, showing the atom-labeling scheme. Displacement ellipsoids are drawn at the 50% probability level.

**Fig. 2.**
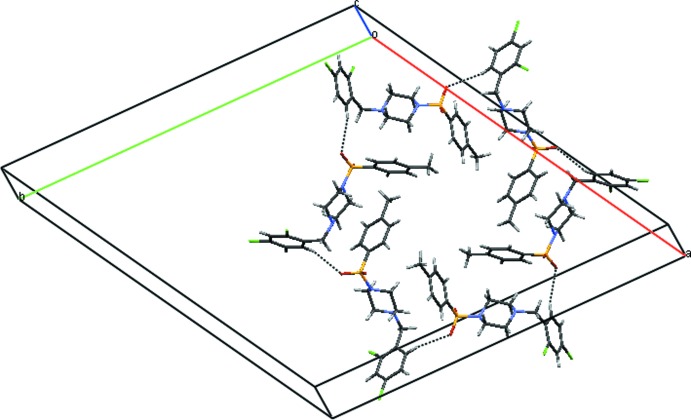
Molecular packing in the title compound displaying R66(60) rings.

**Fig. 3.**
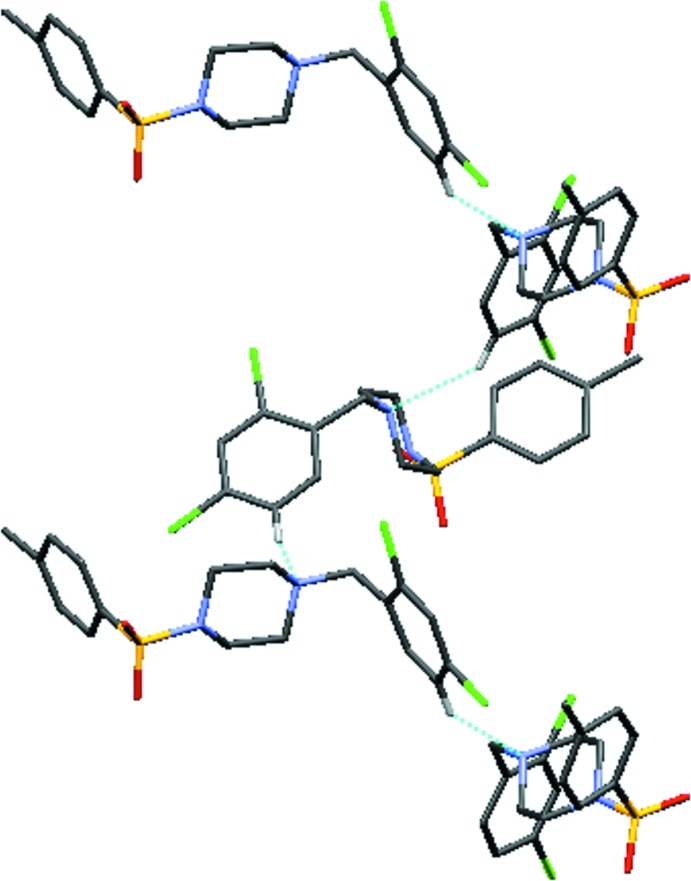
Molecular packing in the title compound displaying C(4) chains.

## supplementary crystallographic information

### Comment

As a part of our continued efforts to study the crystal structures of *N*-(aryl)(4-tosylpiperazin-1-yl)methanone derivatives (Sreenivasa *et al.*, 2013*a*,*b*), we report herein the crystal structure of the title compound.

In the title compound, Fig. 1, the piperazine ring adopts a chair conformation. The dihedral angle between the sulfonyl-bound benzene ring and the best fit plane through the six non-H atoms of the piperazine ring is 72.22 (12)°, while those between the dichlorobenzene and sulfonyl rings and the dichlorobenzene and piperazine rings are 2.44 (13) and 74.16 (2)° respectively. In the crystal, molecules are connected through weak C18—H18···O2 interactions into a hexameric unit generating a R_6_^6^(60) motif, Fig. 2 and Table 1. The molecules are also connected into C(4) chains through a weak C17—H17···N2 interaction, Fig. 3 and Table 1.

### Experimental

A mixture of 1-tosylpiperazine (0.01 mmol), potassium carbonate (0.03 mmol) and 2,4-dichlorobenzyl bromide (0.01 mmol) was added into dry acetonitrile (5 ml). The mixture was stirred at 85°C for 8 h. The reaction was monitored by TLC. Solvent was removed by vacuum distillation and the crude product obtained was purified by column chromatography using 230–400 silica gel and petroleum ether/ethyl acetate as eluent. Colourless prisms were obtained from a mixture of dichloromethane/methanol (7:3) by slow evaporation.

### Refinement

All H atoms were included in calculated positions with C—H bond distances 0.93–0.97 Å and refined in a riding model approximation with *U*_iso_(H) = 1.2–1.5*U*_eq_(C). The solvent used to grow the crystal was a mixture of dichloromethane and methanol. But the resulting electron density was largely uninterpretable. It was decided to model it with the SQUEEZE routine in PLATON (Spek, 2009); more details are given in "_platon_squeeze_details".

### Crystal data

**Table:**

C_18_H_20_Cl_2_N_2_O_2_S	*D*_x_ = 1.294 Mg m^−^^3^
*M**_r_* = 399.32	Melting point: 423 K
Trigonal, *R*3	Mo *K*α radiation, λ = 0.71073 Å
Hall symbol: -R 3	Cell parameters from 2637 reflections
*a* = 28.2896 (5) Å	θ = 2.5–25°
*c* = 13.3041 (3) Å	µ = 0.43 mm^−^^1^
*V* = 9220.8 (3) Å^3^	*T* = 296 K
*Z* = 18	Prism, colourless
*F*(000) = 3744	0.31 × 0.23 × 0.19 mm
Prism	

### Data collection

**Table:**

Bruker APEXII diffractometer	*R*_int_ = 0.033
Radiation source: fine-focus sealed tube	θ_max_ = 25.0°, θ_min_ = 2.5°
Graphite monochromator	*h* = −31→33
phi and ω scans	*k* = −29→28
15194 measured reflections	*l* = −15→15
3596 independent reflections	3608 standard reflections every 22 reflections
2637 reflections with *I* > 2σ(*I*)	intensity decay: 1.0%

### Refinement

**Table:**

Refinement on *F*^2^	Primary atom site location: structure-invariant direct methods
Least-squares matrix: full	Secondary atom site location: difference Fourier map
*R*[*F*^2^ > 2σ(*F*^2^)] = 0.038	Hydrogen site location: inferred from neighbouring sites
*wR*(*F*^2^) = 0.109	H-atom parameters constrained
*S* = 0.95	*w* = 1/[σ^2^(*F*_o_^2^) + (0.0587*P*)^2^ + 6.8101*P*] where *P* = (*F*_o_^2^ + 2*F*_c_^2^)/3
3596 reflections	(Δ/σ)_max_ = 0.089
227 parameters	Δρ_max_ = 0.20 e Å^−^^3^
0 restraints	Δρ_min_ = −0.25 e Å^−^^3^
0 constraints	

### Special details

**Table:**

Geometry. All s.u.'s (except the s.u. in the dihedral angle between two l.s. planes) are estimated using the full covariance matrix. The cell s.u.'s are taken into account individually in the estimation of s.u.'s in distances, angles and torsion angles; correlations between s.u.'s in cell parameters are only used when they are defined by crystal symmetry. An approximate (isotropic) treatment of cell s.u.'s is used for estimating s.u.'s involving l.s. planes.
Refinement. Refinement of *F*^2^ against ALL reflections. The weighted *R*-factor *wR* and goodness of fit *S* are based on *F*^2^, conventional *R*-factors *R* are based on *F*, with *F* set to zero for negative *F*^2^. The threshold expression of *F*^2^ > 2σ(*F*^2^) is used only for calculating *R*-factors(gt) *etc*. and is not relevant to the choice of reflections for refinement. *R*-factors based on *F*^2^ are statistically about twice as large as those based on *F*, and *R*- factors based on ALL data will be even larger.

### Fractional atomic coordinates and isotropic or equivalent isotropic displacement parameters (Å^2^)

**Table:**

	*x*	*y*	*z*	*U*_iso_*/*U*_eq_	
C1	0.64880 (8)	0.09435 (8)	0.76974 (16)	0.0464 (5)	
O2	0.62445 (6)	−0.00584 (6)	0.79558 (13)	0.0674 (5)	
N2	0.44379 (7)	−0.01492 (7)	0.71771 (13)	0.0488 (4)	
C2	0.65949 (9)	0.14258 (9)	0.81461 (18)	0.0567 (6)	
H2	0.6452	0.1423	0.8777	0.068*	
N1	0.54532 (7)	0.00797 (7)	0.79761 (12)	0.0458 (4)	
C3	0.69137 (10)	0.19115 (10)	0.7653 (2)	0.0654 (7)	
H3	0.6984	0.2236	0.7959	0.079*	
C4	0.71321 (9)	0.19286 (10)	0.67143 (19)	0.0595 (6)	
C5	0.70225 (9)	0.14399 (11)	0.62852 (19)	0.0624 (6)	
H5	0.7168	0.1442	0.5657	0.075*	
C6	0.67040 (9)	0.09499 (10)	0.67628 (17)	0.0563 (6)	
H6	0.6634	0.0625	0.6459	0.068*	
O1	0.61109 (6)	0.04263 (7)	0.93700 (12)	0.0678 (5)	
C7	0.74895 (12)	0.24614 (12)	0.6180 (2)	0.0928 (9)	
H7A	0.7857	0.2527	0.6149	0.139*	
H7B	0.7485	0.2753	0.6541	0.139*	
H7C	0.7355	0.2443	0.5511	0.139*	
C8	0.51725 (8)	0.03596 (9)	0.83632 (18)	0.0530 (6)	
H8A	0.5293	0.0698	0.8001	0.064*	
H8B	0.5258	0.0445	0.9069	0.064*	
C9	0.45666 (9)	−0.00078 (9)	0.82310 (16)	0.0531 (6)	
H9A	0.4444	−0.0337	0.8624	0.064*	
H9B	0.4377	0.0176	0.8471	0.064*	
C10	0.47094 (8)	−0.04358 (9)	0.68121 (17)	0.0522 (5)	
H10A	0.4615	−0.0535	0.6113	0.063*	
H10B	0.4588	−0.0769	0.7196	0.063*	
C11	0.53211 (8)	−0.00766 (9)	0.69135 (16)	0.0538 (6)	
H11A	0.5502	−0.0272	0.6688	0.065*	
H11B	0.5447	0.0247	0.6501	0.065*	
C12	0.38530 (9)	−0.04161 (9)	0.69488 (18)	0.0571 (6)	
H12A	0.3804	−0.0505	0.6239	0.068*	
H12B	0.3731	−0.0156	0.7069	0.068*	
C13	0.34892 (8)	−0.09302 (9)	0.75381 (16)	0.0475 (5)	
C14	0.33447 (8)	−0.14556 (9)	0.72265 (16)	0.0492 (5)	
C15	0.30393 (8)	−0.19090 (9)	0.78093 (17)	0.0545 (6)	
H15	0.2957	−0.2253	0.7586	0.065*	
C16	0.28575 (9)	−0.18448 (9)	0.87298 (17)	0.0556 (6)	
C17	0.29689 (9)	−0.13381 (10)	0.90606 (18)	0.0600 (6)	
H17	0.2837	−0.1298	0.9676	0.072*	
C18	0.32797 (9)	−0.08920 (10)	0.84600 (18)	0.0565 (6)	
H18	0.3353	−0.0550	0.8681	0.068*	
S1	0.60861 (2)	0.03176 (2)	0.83193 (4)	0.05200 (18)	
Cl1	0.35347 (3)	−0.15637 (3)	0.60399 (5)	0.0748 (2)	
Cl2	0.24812 (3)	−0.24131 (3)	0.94814 (6)	0.0875 (3)	

### Atomic displacement parameters (Å^2^)

**Table:**

	*U*^11^	*U*^22^	*U*^33^	*U*^12^	*U*^13^	*U*^23^
C1	0.0379 (11)	0.0542 (13)	0.0499 (12)	0.0250 (10)	−0.0040 (9)	−0.0026 (10)
O2	0.0607 (10)	0.0627 (10)	0.0950 (13)	0.0431 (9)	−0.0010 (9)	0.0009 (9)
N2	0.0427 (10)	0.0504 (10)	0.0573 (11)	0.0264 (9)	−0.0001 (8)	0.0044 (8)
C2	0.0525 (14)	0.0616 (15)	0.0552 (14)	0.0278 (12)	0.0022 (11)	−0.0055 (12)
N1	0.0436 (10)	0.0480 (10)	0.0497 (10)	0.0258 (8)	−0.0012 (8)	−0.0079 (8)
C3	0.0589 (15)	0.0552 (15)	0.0773 (18)	0.0248 (12)	−0.0085 (13)	−0.0102 (13)
C4	0.0424 (13)	0.0638 (16)	0.0652 (16)	0.0213 (12)	−0.0084 (11)	0.0070 (12)
C5	0.0506 (14)	0.0795 (18)	0.0567 (15)	0.0321 (13)	0.0024 (11)	0.0042 (13)
C6	0.0487 (13)	0.0640 (15)	0.0590 (14)	0.0303 (12)	−0.0012 (11)	−0.0053 (12)
O1	0.0661 (11)	0.0780 (11)	0.0511 (10)	0.0297 (9)	−0.0086 (8)	0.0039 (8)
C7	0.080 (2)	0.078 (2)	0.096 (2)	0.0213 (16)	−0.0027 (17)	0.0193 (17)
C8	0.0502 (13)	0.0524 (13)	0.0600 (14)	0.0282 (11)	0.0020 (10)	−0.0105 (10)
C9	0.0504 (13)	0.0571 (14)	0.0604 (14)	0.0334 (11)	0.0053 (11)	−0.0067 (11)
C10	0.0494 (13)	0.0582 (13)	0.0500 (13)	0.0278 (11)	−0.0022 (10)	−0.0078 (10)
C11	0.0476 (13)	0.0632 (14)	0.0524 (13)	0.0290 (11)	0.0029 (10)	−0.0101 (11)
C12	0.0515 (13)	0.0636 (15)	0.0633 (15)	0.0341 (12)	−0.0051 (11)	0.0081 (11)
C13	0.0352 (11)	0.0572 (13)	0.0537 (13)	0.0259 (10)	−0.0056 (9)	−0.0021 (10)
C14	0.0367 (11)	0.0631 (14)	0.0469 (12)	0.0242 (11)	−0.0057 (9)	−0.0107 (10)
C15	0.0463 (12)	0.0504 (13)	0.0595 (14)	0.0186 (11)	−0.0053 (10)	−0.0138 (11)
C16	0.0415 (12)	0.0544 (14)	0.0569 (14)	0.0133 (11)	−0.0004 (10)	−0.0044 (11)
C17	0.0506 (13)	0.0647 (15)	0.0569 (14)	0.0230 (12)	0.0089 (11)	−0.0115 (12)
C18	0.0464 (13)	0.0557 (14)	0.0701 (16)	0.0276 (11)	0.0028 (11)	−0.0116 (12)
S1	0.0478 (3)	0.0554 (4)	0.0567 (4)	0.0288 (3)	−0.0042 (2)	0.0013 (3)
Cl1	0.0692 (4)	0.0910 (5)	0.0524 (4)	0.0312 (4)	0.0014 (3)	−0.0202 (3)
Cl2	0.0864 (5)	0.0670 (4)	0.0774 (5)	0.0146 (4)	0.0190 (4)	0.0064 (3)

### Geometric parameters (Å, º)

**Table:**

C1—C2	1.377 (3)	C8—H8A	0.9700
C1—C6	1.382 (3)	C8—H8B	0.9700
C1—S1	1.760 (2)	C9—H9A	0.9700
O2—S1	1.4294 (16)	C9—H9B	0.9700
N2—C9	1.454 (3)	C10—C11	1.512 (3)
N2—C10	1.451 (3)	C10—H10A	0.9700
N2—C12	1.467 (3)	C10—H10B	0.9700
C2—C3	1.375 (3)	C11—H11A	0.9700
C2—H2	0.9300	C11—H11B	0.9700
N1—C8	1.467 (2)	C12—C13	1.514 (3)
N1—C11	1.473 (3)	C12—H12A	0.9700
N1—S1	1.6317 (17)	C12—H12B	0.9700
C3—C4	1.384 (3)	C13—C18	1.390 (3)
C3—H3	0.9300	C13—C14	1.393 (3)
C4—C5	1.380 (3)	C14—C15	1.373 (3)
C4—C7	1.508 (3)	C14—Cl1	1.743 (2)
C5—C6	1.374 (3)	C15—C16	1.375 (3)
C5—H5	0.9300	C15—H15	0.9300
C6—H6	0.9300	C16—C17	1.377 (3)
O1—S1	1.4255 (17)	C16—Cl2	1.734 (2)
C7—H7A	0.9600	C17—C18	1.376 (3)
C7—H7B	0.9600	C17—H17	0.9300
C7—H7C	0.9600	C18—H18	0.9300
C8—C9	1.506 (3)		
			
C2—C1—C6	120.0 (2)	N2—C10—C11	110.00 (17)
C2—C1—S1	120.28 (17)	N2—C10—H10A	109.7
C6—C1—S1	119.69 (17)	C11—C10—H10A	109.7
C9—N2—C10	110.30 (16)	N2—C10—H10B	109.7
C9—N2—C12	113.84 (17)	C11—C10—H10B	109.7
C10—N2—C12	114.78 (17)	H10A—C10—H10B	108.2
C3—C2—C1	119.5 (2)	N1—C11—C10	108.70 (17)
C3—C2—H2	120.2	N1—C11—H11A	109.9
C1—C2—H2	120.2	C10—C11—H11A	109.9
C8—N1—C11	112.00 (16)	N1—C11—H11B	110.0
C8—N1—S1	117.23 (13)	C10—C11—H11B	110.0
C11—N1—S1	116.99 (13)	H11A—C11—H11B	108.3
C2—C3—C4	121.6 (2)	N2—C12—C13	115.93 (17)
C2—C3—H3	119.2	N2—C12—H12A	108.3
C4—C3—H3	119.2	C13—C12—H12A	108.3
C5—C4—C3	117.7 (2)	N2—C12—H12B	108.3
C5—C4—C7	120.6 (2)	C13—C12—H12B	108.3
C3—C4—C7	121.7 (3)	H12A—C12—H12B	107.4
C6—C5—C4	121.7 (2)	C18—C13—C14	115.9 (2)
C6—C5—H5	119.2	C18—C13—C12	119.6 (2)
C4—C5—H5	119.2	C14—C13—C12	124.5 (2)
C5—C6—C1	119.5 (2)	C15—C14—C13	122.7 (2)
C5—C6—H6	120.3	C15—C14—Cl1	116.92 (17)
C1—C6—H6	120.3	C13—C14—Cl1	120.33 (17)
C4—C7—H7A	109.5	C14—C15—C16	118.8 (2)
C4—C7—H7B	109.5	C14—C15—H15	120.6
H7A—C7—H7B	109.5	C16—C15—H15	120.6
C4—C7—H7C	109.5	C15—C16—C17	121.1 (2)
H7A—C7—H7C	109.5	C15—C16—Cl2	119.20 (18)
H7B—C7—H7C	109.5	C17—C16—Cl2	119.73 (18)
N1—C8—C9	108.85 (16)	C18—C17—C16	118.5 (2)
N1—C8—H8A	109.9	C18—C17—H17	120.7
C9—C8—H8A	109.9	C16—C17—H17	120.7
N1—C8—H8B	109.9	C17—C18—C13	122.9 (2)
C9—C8—H8B	109.9	C17—C18—H18	118.6
H8A—C8—H8B	108.3	C13—C18—H18	118.6
N2—C9—C8	110.16 (17)	O1—S1—O2	119.49 (10)
N2—C9—H9A	109.6	O1—S1—N1	106.79 (9)
C8—C9—H9A	109.6	O2—S1—N1	106.62 (9)
N2—C9—H9B	109.6	O1—S1—C1	107.86 (10)
C8—C9—H9B	109.6	O2—S1—C1	107.72 (10)
H9A—C9—H9B	108.1	N1—S1—C1	107.89 (9)
			
C6—C1—C2—C3	0.5 (3)	C12—C13—C14—C15	176.15 (19)
S1—C1—C2—C3	179.42 (17)	C18—C13—C14—Cl1	174.77 (15)
C1—C2—C3—C4	−0.1 (3)	C12—C13—C14—Cl1	−5.6 (3)
C2—C3—C4—C5	−0.4 (3)	C13—C14—C15—C16	1.5 (3)
C2—C3—C4—C7	−179.0 (2)	Cl1—C14—C15—C16	−176.77 (17)
C3—C4—C5—C6	0.6 (3)	C14—C15—C16—C17	1.2 (3)
C7—C4—C5—C6	179.1 (2)	C14—C15—C16—Cl2	−178.92 (16)
C4—C5—C6—C1	−0.2 (3)	C15—C16—C17—C18	−1.7 (4)
C2—C1—C6—C5	−0.3 (3)	Cl2—C16—C17—C18	178.44 (18)
S1—C1—C6—C5	−179.28 (17)	C16—C17—C18—C13	−0.5 (3)
C11—N1—C8—C9	57.6 (2)	C14—C13—C18—C17	2.9 (3)
S1—N1—C8—C9	−163.12 (15)	C12—C13—C18—C17	−176.7 (2)
C10—N2—C9—C8	60.6 (2)	C8—N1—S1—O1	44.09 (18)
C12—N2—C9—C8	−168.74 (17)	C11—N1—S1—O1	−178.60 (15)
N1—C8—C9—N2	−58.1 (2)	C8—N1—S1—O2	172.90 (15)
C9—N2—C10—C11	−60.5 (2)	C11—N1—S1—O2	−49.80 (17)
C12—N2—C10—C11	169.35 (18)	C8—N1—S1—C1	−71.63 (17)
C8—N1—C11—C10	−57.5 (2)	C11—N1—S1—C1	65.67 (17)
S1—N1—C11—C10	163.11 (15)	C2—C1—S1—O1	−23.1 (2)
N2—C10—C11—N1	57.9 (2)	C6—C1—S1—O1	155.79 (17)
C9—N2—C12—C13	−56.4 (2)	C2—C1—S1—O2	−153.37 (17)
C10—N2—C12—C13	72.1 (2)	C6—C1—S1—O2	25.6 (2)
N2—C12—C13—C18	88.3 (2)	C2—C1—S1—N1	91.89 (18)
N2—C12—C13—C14	−91.3 (3)	C6—C1—S1—N1	−89.19 (18)
C18—C13—C14—C15	−3.4 (3)		

### Hydrogen-bond geometry (Å, º)

**Table:**

*D*—H···*A*	*D*—H	H···*A*	*D*···*A*	*D*—H···*A*
C18—H18···O2^i^	0.93	2.70	3.575 (3)	157
C17—H17···N2^ii^	0.93	2.70	3.485 (3)	143

Symmetry codes: (i) *y*+1/3, −*x*+*y*+2/3, −*z*+5/3; (ii) −*x*+*y*+2/3, −*x*+1/3, *z*+1/3.
